# Early and late stage MPN patients show distinct gene expression profiles in CD34^+^ cells

**DOI:** 10.1007/s00277-021-04615-8

**Published:** 2021-08-14

**Authors:** Julian Baumeister, Tiago Maié, Nicolas Chatain, Lin Gan, Barbora Weinbergerova, Marcelo A. S. de Toledo, Jörg Eschweiler, Angela Maurer, Jiri Mayer, Blanka Kubesova, Zdenek Racil, Andreas Schuppert, Ivan Costa, Steffen Koschmieder, Tim H. Brümmendorf, Deniz Gezer

**Affiliations:** 1 Department of Hematology, Oncology, Hemostaseology, and Stem Cell Transplantation, Faculty of Medicine, RWTH Aachen University, Aachen, Germany; 2 Center for Integrated Oncology, Aachen Bonn Cologne Duesseldorf (CIO ABCD), Aachen, Germany; 3 Institute for Computational Genomics, RWTH Aachen University, Aachen, Germany; 4 IZKF Genomics Core Facility, RWTH Aachen University Medical School, Aachen, Germany; 5 Department of Internal Medicine, Hematology and Oncology, Masaryk University and University Hospital Brno, Brno, Czech Republic; 6 Department of Orthopedic Surgery, University Hospital RWTH Aachen, Aachen, Germany; 7 Institute of Hematology and Blood Transfusion, Prague, Czech Republic; 8 Joint Research Center for Computational Biomedicine, RWTH Aachen, Aachen, Germany

**Keywords:** MPN, Gene expression, JAK2V617F, CD34

## Abstract

Myeloproliferative neoplasms (MPN), comprising essential thrombocythemia (ET), polycythemia vera (PV), and primary myelofibrosis (PMF), are hematological disorders of the myeloid lineage characterized by hyperproliferation of mature blood cells. The prediction of the clinical course and progression remains difficult and new therapeutic modalities are required. We conducted a CD34^+^ gene expression study to identify signatures and potential biomarkers in the different MPN subtypes with the aim to improve treatment and prevent the transformation from the rather benign chronic state to a more malignant aggressive state. We report here on a systematic gene expression analysis (GEA) of CD34^+^ peripheral blood or bone marrow cells derived from 30 patients with MPN including all subtypes (ET (*n* = 6), PV (*n* = 11), PMF (*n* = 9), secondary MF (SMF; post-ET-/post-PV-MF; *n* = 4)) and six healthy donors. GEA revealed a variety of differentially regulated genes in the different MPN subtypes vs. controls, with a higher number in PMF/SMF (200/272 genes) than in ET/PV (132/121). PROGENγ analysis revealed significant induction of TNFα/NF-κB signaling (particularly in SMF) and reduction of estrogen signaling (PMF and SMF). Consistently, inflammatory GO terms were enriched in PMF/SMF, whereas RNA splicing–associated biological processes were downregulated in PMF. Differentially regulated genes that might be utilized as diagnostic/prognostic markers were identified, such as *AREG*, *CYBB*, *DNTT*, *TIMD4*, *VCAM1*, and S100 family members (*S100A4/8/9/10/12*). Additionally, 98 genes (including *CLEC1B*, *CMTM5*, *CXCL8*, *DACH1*, and *RADX*) were deregulated solely in SMF and may be used to predict progression from early to late stage MPN.

**Graphical abstract:**

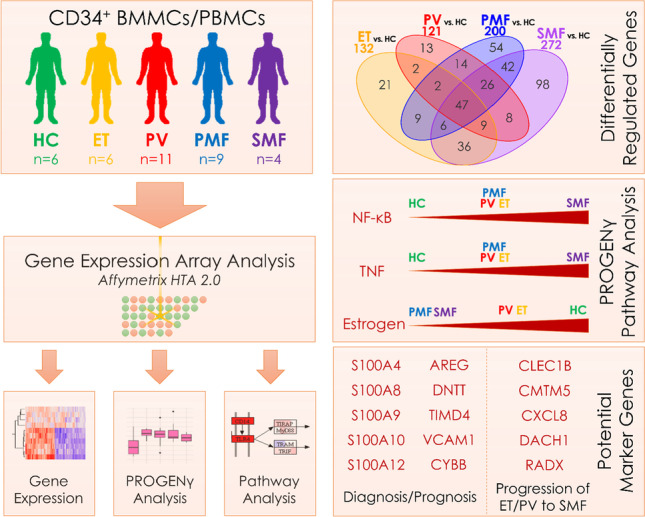

**Supplementary Information:**

The online version contains supplementary material available at 10.1007/s00277-021-04615-8.

## Introduction

Classical Philadelphia chromosome-negative (Ph^−^) myeloproliferative neoplasms (MPN) can be subdivided into essential thrombocythemia (ET), polycythemia vera (PV), and primary myelofibrosis (PMF). Although clinically distinct, they typically share mutations in J*AK2*, *CALR*, or *MPL*, or are termed triple-negative in the absence of these mutations [1–3]. *JAK2*V617F is the most abundant mutation, present in over 90% of PV, and 55–65% of ET and PMF patients [4].

Although the modified WHO diagnostic criteria include the presence of one of the abovementioned mutations as major criteria, cooperating mutations in other genes implicated in epigenetic regulation, transcriptional regulation, mRNA splicing, and signal transduction are often observed in PMF or SMF [5]. The impact of these mutations that are also associated with disease progression has been incorporated into prognostic scores such as MIPSS70 [6]. Furthermore, it was reported that some MPN courses are more dependent on non-classical driver mutations, and this might play a major role in transformation [7]. Despite specific subtypes and driver mutations, the clinical course varies among MPN patients, but the underlying mechanisms for this variability are still not fully understood.

The identification of gene expression profiles and biomarkers that enable the prediction of transition from a rather “benign” (i.e., mostly characterized on the cellular level by increased proliferation) chronic to a more malignant aggressive state (defined by the acquisition of a differentiation block or induction of fibrotic transformation) is important in order to develop targeted therapeutic strategies and prevent hematological progression to secondary myelofibrosis (SMF) or transformation to acute myeloid leukemia (AML). Among the Ph^−^ MPN subtypes, ET and PV are considered early stage MPN. They are typically characterized by a relatively indolent disease course, as are some prefibrotic PMF. However, they all bare the risk of thromboembolic complications and eventually, progression into SMF and/or AML. Consequently, overt PMF and SMF are considered late stage MPN [8].

Treatment with the tyrosine-kinase inhibitor ruxolitinib is an example for rational therapy by targeting the essential pro-tumorigenic mutated JAK2 [9]. Ruxolitinib confers clinical benefit by reducing splenomegaly and other disease-related symptoms, but largely fails to induce complete molecular remission [10]. Therapeutic strategies in MPN are based on clinical conditions and laboratory results; only recently have molecular findings been integrated into risk progression models [11]. The identification of new targetable molecular components might result in further improvement of risk stratification as well as targeted therapeutic strategies. Genome-wide gene expression analysis (GEA) is a promising approach to resolve these targets.

Several GEA studies of patient-derived CD34^+^ cells have been conducted to investigate deregulated gene expression in MPN [12–17]. However, not all disease entities were included, thereby impeding a direct comparison of the different entities among themselves and/or their normal counterparts. Therefore, we conducted gene expression profiling of CD34^+^ purified bone marrow (BM)– or peripheral blood (PB)–derived cells from ET, PV, PMF, and SMF patients and compared them to CD34^+^ cells from healthy controls (HC).

## Methods

### Patient samples

PB and BM samples from MPN patients were obtained at the Department of Hematology, Oncology, Hemostaseology, and Stem Cell Transplantation at Uniklinik RWTH Aachen after written informed consent and approval of the local ethics committee (EK127/12). Control CD34^+^ cells from PB of individual healthy donors were obtained from Lonza. Unfractionated BM samples from JAK2V617F-positive untreated MPN patients within the CELL project — MPN database (MIND) — after written informed consent were provided from the Department of Internal Medicine, Hematology and Oncology of Brno University Hospital, Czech Republic. Femoral heads were obtained from the Department of Orthopedics from the Uniklinik RWTH Aachen, each after written informed consent and in compliance with the local ethics committee (EK300/13).

### Next-generation sequencing

Relevant coding regions of 32 genes associated with hematologic malignancies were analyzed using an amplicon-based next-generation sequencing (NGS) panel (Truseq Custom Amplicon Kit, Illumina) as previously described [18]. Allele burdens (AB) were determined accordingly. Variants were called with a bidirectional frequency of > 5% (JAK2V617F and KITD816V > 1%) and reviewed manually.

### Isolation of CD34^+^ cells

PB and BM MNCs (PBMCs/BMMCs) from MPN patients obtained from the Department of Hematology, Oncology, Hemostaseology, and Stem Cell Transplantation at Uniklinik RWTH Aachen were isolated with Ficoll-Paque Premium (GE Healthcare). Enrichment of the CD34^+^ cell population was performed by magnetic cell separation (MACS) with the CD34 MicroBead Kit (Miltenyi) following the manufacturer’s instructions. The purity of CD34^+^ cells was assessed by flow cytometry using CD34-FITC and CD45-APC antibodies (BD Biosciences). Samples with a purity of > 50% were used. Additional patient data is given in Supplemental Table 1–2.

### RNA isolation and RT-qPCR

RNA was isolated with the RNeasy Micro kit (Qiagen). RT-qPCR was performed as described by Czech et al. [19]. Primers are listed in Supplemental Table 3.

### Gene expression analysis

Genome-wide transcriptome analysis was performed using the Affymetrix HTA 2.0 platform (Thermo Fisher Scientific). 6.6 ng total RNA from each sample was prepared and hybridized to the HTA 2.0 arrays according to the WT Pico kit manual (Thermo Fisher Scientific). Batch correction was applied using sva and Combat [20]. Downstream analysis was performed in R.

### Statistical analysis

Data were normalized with signal space transformation (SST) in conjunction with robust multiple-array (RMA) average normalization method (SST-RMA) with Affymetrix Expression console. Statistical analysis of RT-qPCR data was performed with the Mann–Whitney *U* test (GraphPad Prism 8).

### Data sharing statement

Microarray data have been deposited at GEO under accession number GSE174060.

## Results

### Gene expression signatures in myelofibrosis patient-derived CD34^+^ cells show more pronounced differences compared to HCs than early stage MPN (ET/PV)

**Fig. 1:**
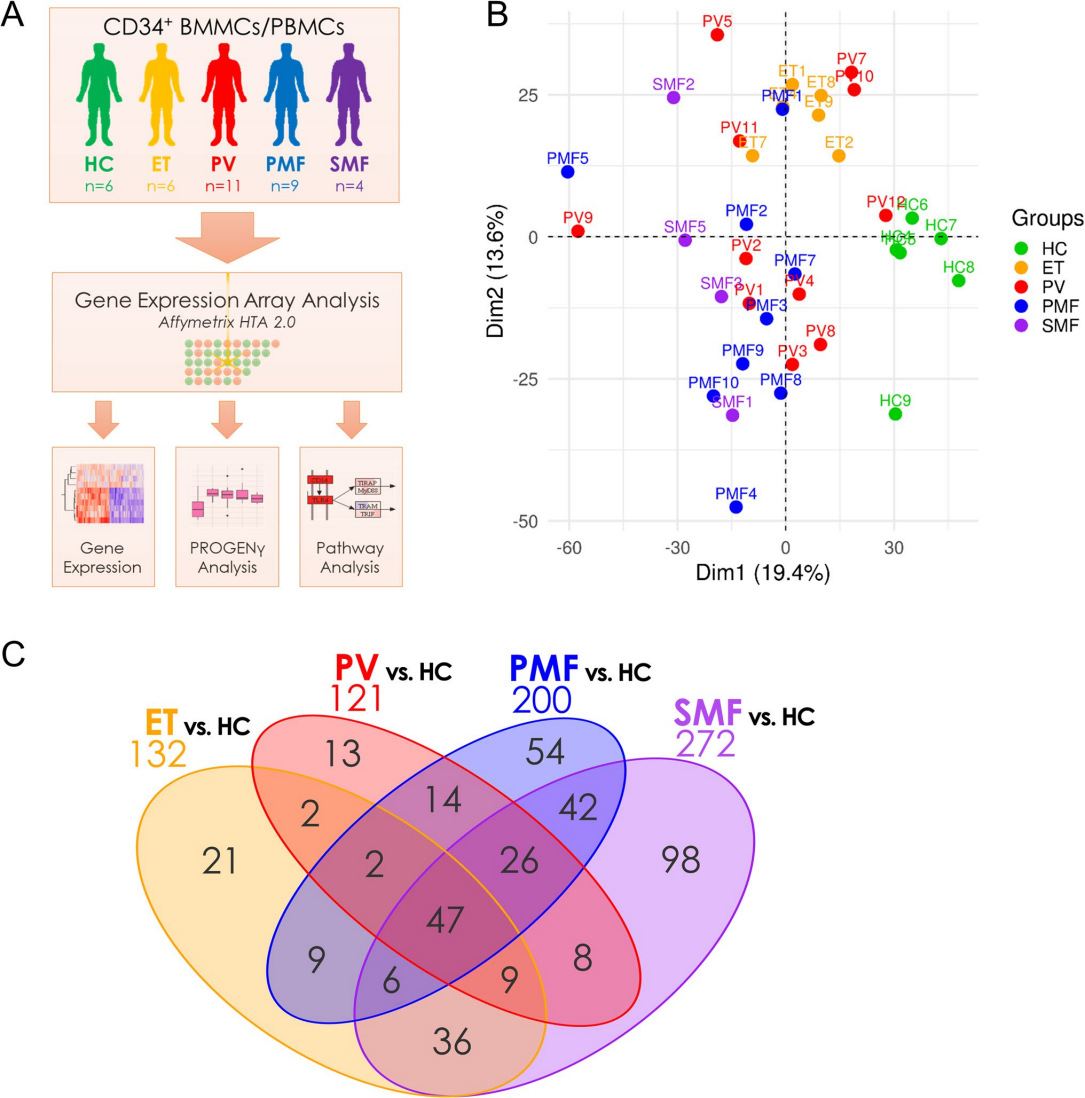
Distinct gene expression profiles of CD34^+^-enriched MNCs from ET, PV, PMF, and SMF patients and HC. **A** Experimental design of the study. Patient-derived BMMCs and PBMCs were isolated from BM aspirations or PB samples by density gradient centrifugation and enriched for the CD34^+^ population by magnetic cell sorting. The isolated RNA was analyzed using the Affymetrix HTA 2.0 platform and analyzed regarding gene expression, Pathway RespOnsive GENes (PROGENy), and enriched KEGG pathways and gene ontologies. **B** Unsupervised principal component analysis (PCA) of gene expression data between the different MPN samples and HCs. **C** Venn diagram of the differentially expressed genes (*p* < 0.05 and |FC|> 1.5). A list of all comparisons is given in Supplemental Table 4. BMMCs, bone marrow mononuclear cells; ET, essential thrombocythemia; HC, healthy controls; MNCs, mononuclear cells; PMF, primary myelofibrosis; PBMCs, peripheral blood mononuclear cells; PV, polycythemia vera; SMF, secondary myelofibrosis

To investigate differences in the transcriptional profile of hematopoietic stem and progenitor cells (HSPCs) derived from ET, PV, PMF, and SMF patients as well as healthy donors, BM- or PB-derived CD34^+^ cells were subjected to GEA (Fig. 1A). The analysis included ET (*n* = 6), PV (*n* = 11), PMF (*n* = 9), and post-ET-MF/post-PV-MF (*n* = 2 each) patients, as well as HCs (*n* = 6; Supplemental Table 1), with *JAK2*V617F- (*n* = 23), *CALR*- (*n* = 4), *MPL*-mutated (*n* = 1), and triple-negative (*n* = 2) patients. The screening for MPN-associated driver and additional mutations was performed by NGS (Supplemental Table 2).

Principal component analysis (PCA) revealed clustering of MPN samples and separation from HC samples (Fig. 1B). While the ET samples clustered in proximity to HCs, PV, PMF, and SMF samples were located more distantly with a broader distribution within the groups. Among the four MPN entities, we observed a continuum in dimension 2 from ET over PV, PMF, to finally SMF (Supplemental Fig. 1A). The distribution of PV, PMF, and SMF samples indicates heterogeneity between the diseases. We observed no clear clustering of the post-ET-MF/post-PV-MF samples or the different driver mutation with PCA (Supplemental Fig. 1B). Interestingly, high AB of the respective driver mutation were correlated with low PC1/PC2 values typically associated with PMF/SMF samples (Supplemental Fig. 1C) and potentially indicates beginning transformation. We analyzed differentially expressed genes compared to HCs and observed that PMF and SMF showed the highest number of differentially regulated genes (200 and 272, resp.), while PV (121) and ET (132) showed lower numbers (Fig. 1C).

Ninety-eight genes were uniquely regulated in SMF vs. HC. These genes might include potential biomarkers for progression from ET or PV to SMF, of which some will be addressed hereinafter. Furthermore, we identified 121 genes that were differentially regulated in both PMF and SMF showing some similarity. In total, 47 genes were differentially regulated in all four entities. A list of these genes and all other comparisons is provided in Supplemental Table 4. Direct comparisons between the MPN subtypes (without HCs) revealed a high degree of similarity between ET/PV and PMF/SMF with few differentially regulated genes (Supplemental Fig. 2).

**Fig. 2:**
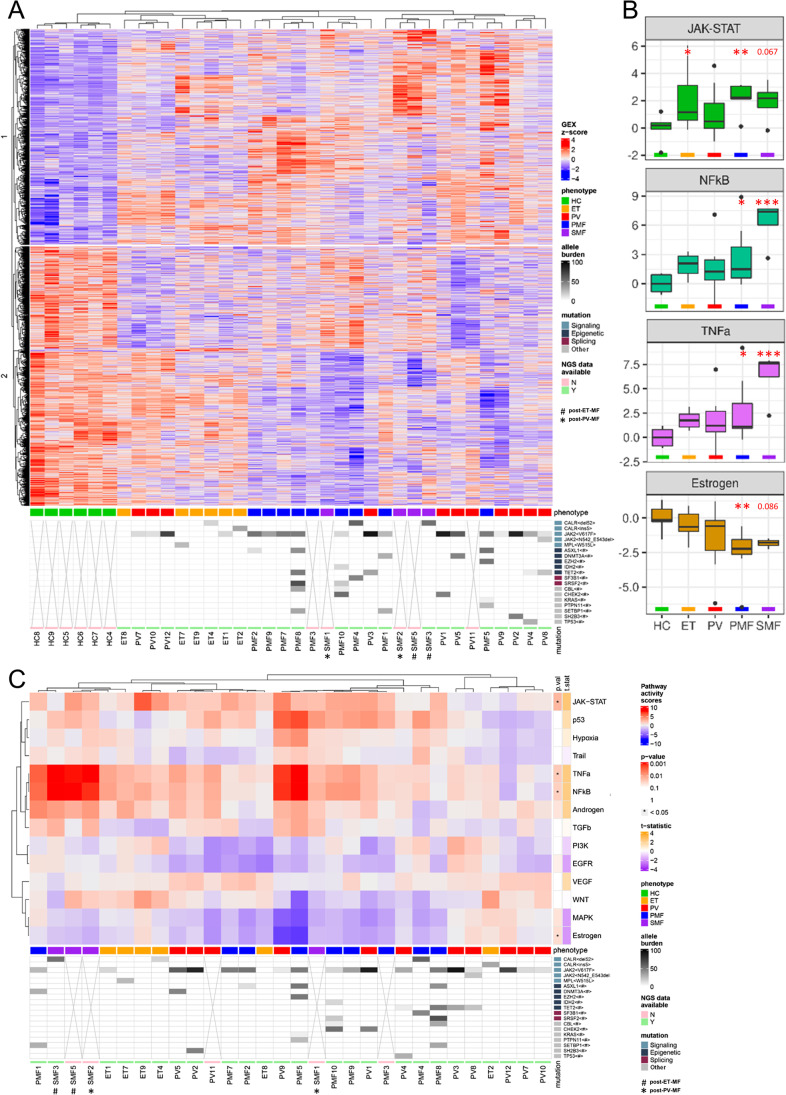
Hierarchical clustering of differentially expressed genes and PROGENγ activity scores in CD34^+^-enriched MNCs from ET, PV, PMF, and SMF patients and HC. **A** Heatmap of unsupervised clustering of all 5815 differentially regulated genes of each MPN subtype vs. control samples. Allele burden of MPN-associated driver and bystander mutations is indicated by shades of gray. Samples lacking NGS data are marked with x. **B** Pathway RespOnsive GENes for activity inference (PROGENγ) analysis of JAK/STAT, estrogen, NF-κB, and TNFα signaling pathways in patients- or healthy donor-derived CD34^+^ cells, arranged by group (HC, ET, PV, PMF, and SMF), normalized in respect to HC. Pathways are restricted to significantly deregulated pathways in all MPN samples vs. HC. An overview of all pathways is given in Supplemental Fig. 3. **C** Two-dimensional unsupervised clustering of PROGENγ activity scores of individual HC, ET, PV, PMF, and SMF samples, normalized in respect to HC. *P*-values and *t*-statistics were calculated by *t*-test (* *p* < .05, ** *p* < .01, *** *p* < .001). ET, essential thrombocythemia; HC, healthy controls; MNCs, mononuclear cells; NGS, next-generation sequencing; PMF, primary myelofibrosis; PV, polycythemia vera; SMF, secondary myelofibrosis; #, post-ET-MF; *, post-PV-MF

To test whether the most significantly regulated genes allowed hierarchical clustering between the disease groups, a heatmap was generated by unsupervised hierarchical clustering (Fig. 2A). All HC samples clustered together. The ET samples grouped together with three PV samples that were located in between and appeared to show more PV-like expression patterns. Most PV samples grouped next to MF samples that also clustered together with three of the four SMF samples (comprising two post-ET-MF/post-PV-MF each) being adjacent to each other. Clustering analysis underlines the paradigm of “early” and “late” stage MPN. The predominant hypothesis that explains how JAK2V617F leads to three different diseases relies on the determining influence of the molecular background and the occurrence of additional mutations [21]. Increased number of concurrent mutations was shown to correlate with a PMF-like phenotype. This is reflected by our NGS analysis, as most patients carrying more than two bystander mutations were PMF patients.

### NF-κB and TNFα pathways are upregulated in CD34^+^ cells in late stages of MPN

After elucidating single gene changes, we focused on PROGENy (Pathway RespOnsive GENes) analysis exploring the activation of cancer pathways using the top 1000 genes according to significance for the model generation [22]. Besides expected upregulation of JAK-STAT signaling, we observed strong upregulation of NF-κB and TNFα signaling compared with HCs, as well as downregulation of estrogen signaling (Fig. 2B–C). Although upregulation of these inflammatory pathways has been described [23], we could show a prominent upregulation in post-PV-MF/post-ET-MF samples.

Since we observed a strong activation of inflammatory pathways, we analyzed NF-κB (Supplemental Fig. 4) and TNFα pathways in more detail (Supplemental Fig. 5), with KEGG pathways representing network maps of molecular interactions. Within canonical and noncanonical NF-κB pathways, *BTK* and *IKKβ* were upregulated, as well as several pro-survival target genes such as *Bcl-XL*, *c-IAP1/2*, and *c-FLIP*, or the inflammatory targets *COX2*, *MIP-1β*, and *VCAM1*. *TNF*, TNF receptors (*TNFR*) 1 and 2, and genes associated with leukocyte recruitment, inflammatory cytokines and mediators, and cell adhesion were also upregulated.

**Fig. 3:**
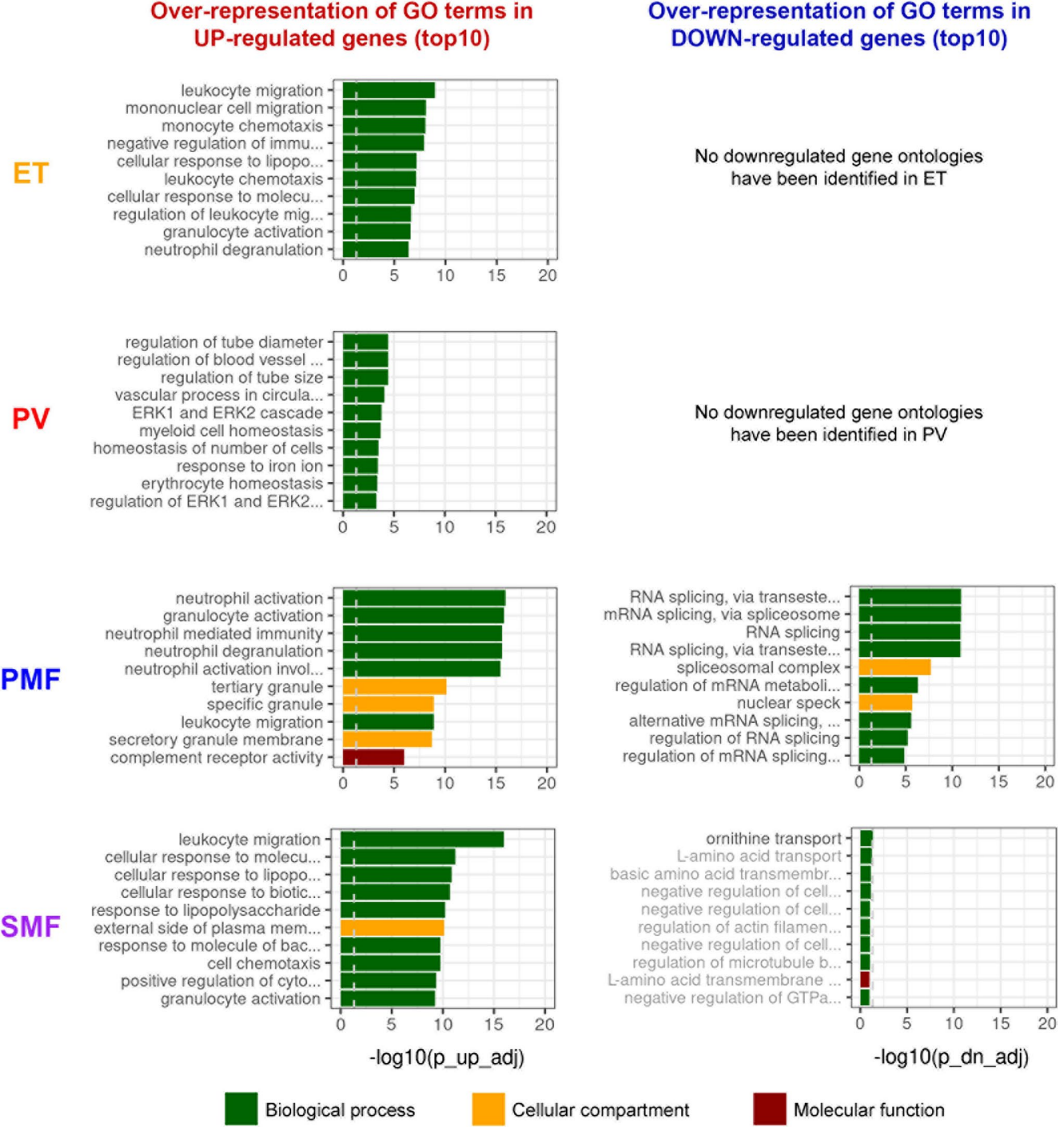
Gene ontology (GO) analysis in MPN patient- vs. HC-derived CD34^+^ cells. GO terms analysis of biological processes (green), and cellular components (yellow), and molecular functions (red). Graphs show the 10 most significantly enriched ontologies with over-representation of GO terms in up- (left) or downregulated (right) genes vs. HC, ranked by adj. *p*-value. GO terms were restricted to terms with less than 500 genes. A list of all deregulated GO terms including gene numbers and *p*-values is provided in Supplemental Table 5. ET, essential thrombocythemia; HC, healthy controls; PMF, primary myelofibrosis; PV, polycythemia vera; SMF, secondary myelofibrosis

To identify deregulated pathways, we examined whether the different subtypes showed differences in gene ontology (GO) analysis compared with HCs. We observed the induction of GOs associated with inflammation such as neutrophil activation, leukocyte migration, acute inflammatory response, and others in ET, PMF, and SMF (Fig. 3, Supplemental Table 5). Surprisingly, in PV, inflammatory responses and leukocyte migration were not as upregulated as in ET. Instead, blood vessel–associated biological processes represented the strongest enriched ontologies in PV. Furthermore, RNA splicing–associated biological processes were downregulated in PMF, but not in ET, PV, and SMF, fitting the high frequency of mutations in RNA splicing–associated genes in PMF [24]. In our study, mutations in *SRSF2* and *SF3B1* in two PMF patients each, but not the other subtypes, were identified by NGS. Furthermore, several genes involved in the cellular response to oxidative stress and NRF2 target genes were upregulated in our investigation (Supplemental Fig. 6). NRF2 is the master regulator of the oxidative stress response by regulating the expression of a variety of antioxidative enzymes [25].

The direct interaction of the clonal with normal HSPCs or endothelial and mesenchymal stromal cells plays a central role in inflammation, thrombosis, and extramedullary hematopoiesis [26]. Therefore, we investigated the expression of cell adhesion molecules and observed an upregulation of *ITGB2*, *ITGB7*, *SELP*, and *SELPLG* in all subtypes. They are involved in leukocyte adhesion and have not been investigated in MPN so far and stand as interesting targets for future studies (Supplemental Fig. 7).

### Differentially regulated potential marker genes in CD34^+^ HSPCs from MPN subtypes

**Fig. 4:**
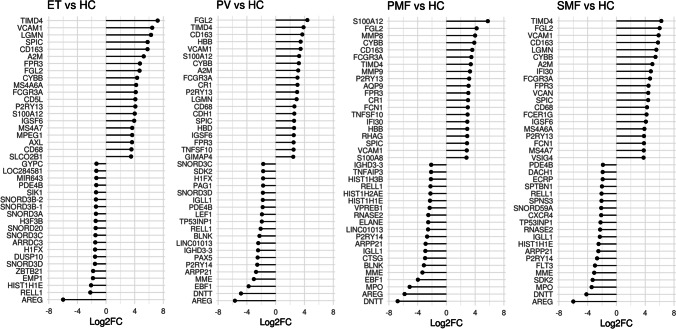
Differentially expressed genes. (A) Top 20 up- and downregulated genes in ET, PV, SMF, and PMF vs. HC (*p* < 0.05), ranked by Log2FC. ET, essential thrombocythemia; HC, healthy controls; PMF, primary myelofibrosis; PV, polycythemia vera; SMF, secondary myelofibrosis

At the single gene level, we have identified the top 20 up- and downregulated genes for each subtype (Fig. 4). *AREG* was prominently downregulated in all entities compared to HCs. *AREG* encodes amphiregulin, a ligand of the epidermal growth factor receptor (EGFR). AREG binding to the EGFR activates major intracellular signaling cascades governing cell survival, proliferation, and motility [27]. On the other hand, it inhibits the growth of certain aggressive carcinoma cell lines and has an immunosuppressive function by facilitating the suppressor capacity of Tregs [28]. *AREG* expression was observed to be downregulated in myeloid and lymphoid neoplasms [29].

*DNTT* was the strongest downregulated gene in PMF and inversely correlated with driver mutation AB in CD34^+^ of all MPN subtypes combined (Supplemental Fig. 8). *DNTT*, which was also found to be downregulated in PV and SMF in our study, is expressed in pre-B and pre-T lymphocytes during early differentiation. It is a diagnostic and classification marker in ALL and low expression correlates with inferior survival [30, 31].

**Fig. 5:**
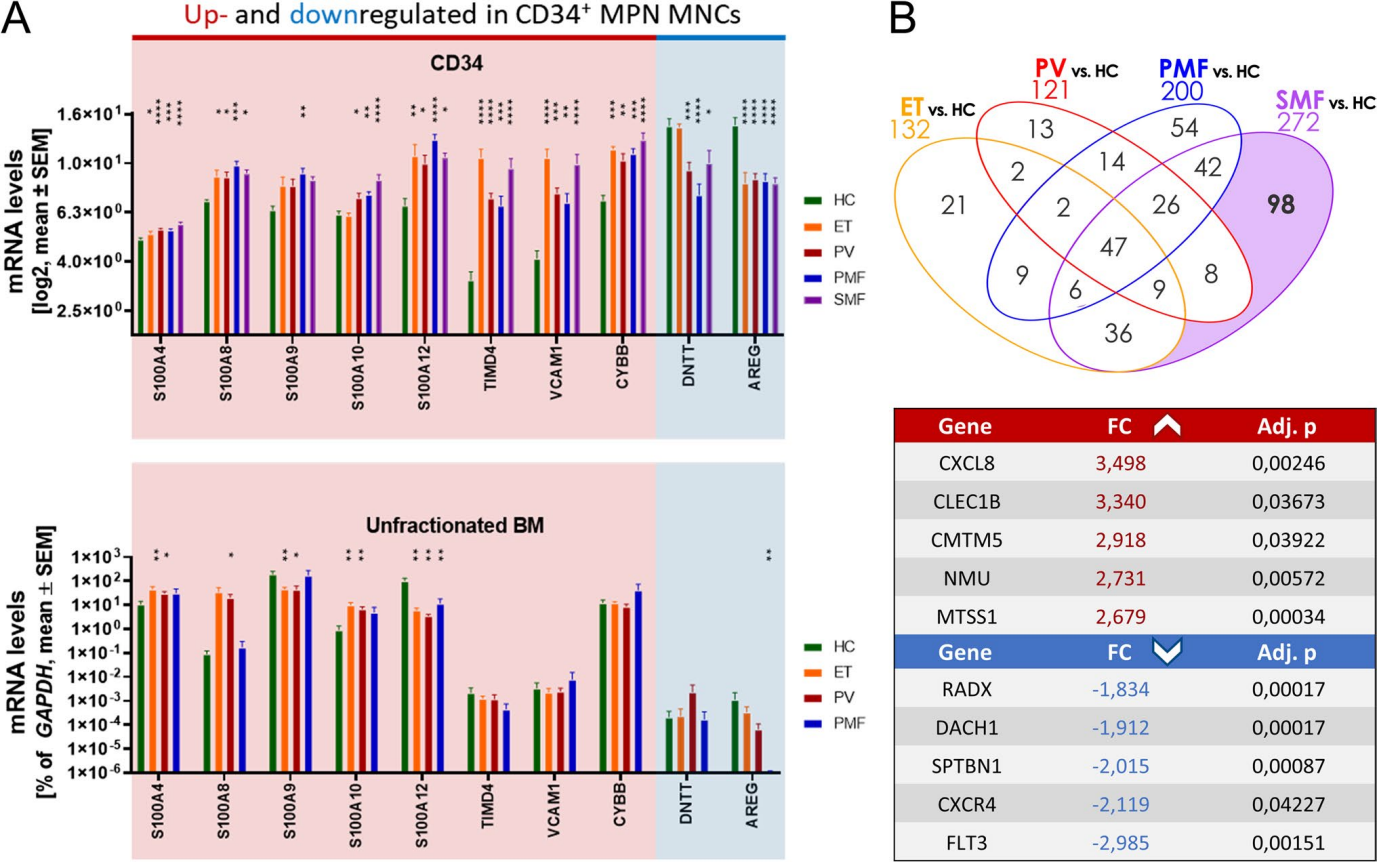
Expression of potential marker genes in CD34^+^ cells compared with unfractionated BM cells. **A** Gene expression levels of S100A4, S100A8, S100A9, S100A10, S100A12, TIMD4, VCAM1, CYBB, DNTT, and AREG in ET, PV, and MF patient- and HC-derived CD34^+^ cells (top; adj. *p*) or unfractionated BMMCs analyzed by RT-qPCR (bottom; *n* = 5 respectively; Mann–Whitney *U* test). **B** Top 10 genes that were differentially regulated in SMF vs. HC, but not the other comparisons, ranked by FC and filtered by adj. *p* < .05. BM, bone marrow; ET, essential thrombocythemia; FC, fold change; HC, healthy controls; MNCs, mononuclear cells; PMF, primary myelofibrosis; PV, polycythemia vera; SMF, secondary myelofibrosis. * *p* < .05, ** *p* < .01, *** *p* < .001, **** *p* < .0001 compared to HC

In addition to these downregulated genes, we also identified the upregulation of *TIMD4*, *VCAM1*, and *CYBB/NOX2* in all subtypes. Furthermore, we observed increased expression of *several S100 genes* in CD34^+^ cells of one or more MPN subtypes (Fig. 5A). S100A4, S100A8, S100A9, and S100A12 are calcium- and zinc-binding proteins that function in the regulation of inflammatory processes and have been found to be associated with myelopoiesis [32]. Increased levels of S100 proteins in MPN patient-derived granulocytes and plasma as well as in PV CD34^+^ cells (*S100A4*, *S100A9*) have previously been reported [33]. In our GEA, we observed strongly elevated levels of *S100A8*, *S100A9*, and *S100A12* in PMF, but also in ET and PV (*S100A8*, *S100A12*). S100A8/9 dimers were reported to promote tumor cell proliferation by creating a pro-inflammatory microenvironment and contribute to disease progression in MPN, whereas tasquinimod, an S100A8/S100A9 inhibitor, inhibited the MPN phenotype [34–36].

### Deregulation of potential marker genes is restricted to the CD34^+^ compartment

As we have observed deregulated expression of the potential marker genes *S100A4*, *S100A8*, *S100A9*, *S100A10*, *S100A12*, *TIMD4*, *VCAM1*, *CYBB*, *DNTT*, and *AREG*, we aimed to assess whether this effect is restricted to the CD34^+^ compartment or also observed in unfractionated BM cells. Therefore, we performed RT-qPCR analysis of a different set of de novo and untreated *JAK2*V617F-positive BMMCs derived from ET, PV, and PMF patients and CD45-enriched HC cells that were isolated from femoral heads during hip replacement (*n* = 5).

In the case of *S100A4*, *S100A8*, *S100A9*, and *S100A10*, unfractionated BM and purified CD34^+^ cells showed some similarities (Fig. 5A). Strikingly, CD34^+^ purified cells from MPN patients showed significant upregulation of *S100A12*, whereas unfractionated BM showed downregulation. In addition to that, the strongly upregulated genes *TIMD4*, *VCAM1*, and *CYBB* only showed significant upregulation in the CD34 purified compartment. In the case of *DNTT*, the significant downregulation was only detected in CD34^+^ cells from PV and MF patients while no changes were observed in ET or in unfractionated BM cells. Similarly, *AREG* showed strong downregulation only in the CD34^+^ fraction of all MPN subtypes and unfractionated BM cells from PMF patients. These results confirm the importance of investigating the CD34^+^ fraction and indicate that *TIMD4*, *VCAM1*, *CYBB*, *DNTT*, *AREG*, and *S100A12* may play a central role in MPN pathophysiology.

With the aim to identify marker genes for the progression from ET or PV to SMF, we focused on genes exclusively deregulated in SMF (Fig. 5B, Supplemental Table 4). The top 5 upregulated genes were *CXCL8*, *CLEC1B*, *CMTM5*, *NMU*, and *MTSS1*. Elevated blood plasma levels of the pro-inflammatory cytokine CXCL8, also termed interleukin 8 (IL-8), have been identified as a negative prognostic indicator in PMF [37]. *CLEC1B* is expressed by myeloid cells and modulates the secretion of cytokines [38]. The neuropeptide encoded by *NMU* plays a role in inflammatory diseases and its overexpression was identified in solid tumors [39]. *MTSS1* and *CMTM5* are tumor suppressor genes [40–42], although in some cancer types, MTSS1 was identified as a tumor enhancer [43, 44]. To our knowledge, an implication of *CLEC1B*, *NMU*, *MTSS1*, and *CMTM5* in Ph^−^ MPN is yet to be described. *CXorf57*, *DACH1*, *SPTBN1*, *CXCR4*, and *FLT3* were identified as the most strongly downregulated genes. *CXorf57* or *RADX* encodes a DNA-binding protein that prevents replication fork collapse by antagonizing the accumulation of RAD51 [45]. *DACH1* was identified as a suppressor of the progression of various neoplasms and its downregulation is associated with a poor prognosis [46]. Reduced *SPTBN1* levels were discovered in colon and pancreatic cancer, potentially contributing to tumor initiation or progression [47]. We are the first group to demonstrate the downregulation of *RADX*, *DACH1*, and *SPTBN1* in Ph^−^ MPN. Reduced expression of *CXCR4* in CD34^+^ cells was recently identified as a potential diagnostic and prognostic biomarker in PMF [48]. Correspondingly, we observed strongly decreased *CXCR4* expression in PMF (− 1.36 fold) and SMF (− 2.12 fold). Surprisingly, *FLT3* was the most downregulated gene, although it plays an important role in the survival and proliferation of HSPCs and contributes to MF [49].

## Discussion

Although the identification of the driver mutations of *JAK2*, *CALR*, and *MPL* found in the majority of MPN (excluding triple-negative cases) has provided substantial insight into the pathogenesis of Ph^−^ MPN, one of the highest unmet clinical needs is the paucity of biomarkers that reliably predict the disease course. The GEA performed within this study aimed to identify differential transcriptional signatures between the MPN entities to provide novel pathogenic insights and new diagnostic or prognostic information. Within Ph^−^ MPN, myelofibrosis (PMF or SMF) differs from ET and PV, both clinically and molecularly, with a higher prevalence of additional mutations and inflammatory changes [5, 50].

In our approach to analyze gene expression in CD34^+^ cells derived from ET, PV, PMF, and SMF patients, we revealed patterns that are clearly distinguishable from those of HCs. Yet, ET samples clustered closest to HCs, underlining their clinically indolent behavior (and the overall almost unimpaired life expectancy of affected patients). The discrimination of the MPN samples was not dependent on the driver mutation, which is underlined by our finding that *CALR-*, *MPL-*mutated, or triple-negative patients did not differ in the PCA or heatmaps from *JAK2*-mutated samples. Within the different MPN subtypes, PV patients showed gene expression patterns like those from ET patients, but overlapping patterns with PMF patients were also identified. In addition, the gene expression patterns of PMF and SMF patients revealed a high degree of similarity, corroborating the paradigm of “early” and “late” stage MPN.

Oncogenic pathway activity analysis using PROGENγ revealed that TNFα signaling and its effector pathway, NF-κB, were upregulated in all MPN subtypes (in line with findings from other groups [17, 23, 51]), but particularly in SMF, suggesting that SMF patients are characterized by a more pronounced inflammatory condition. BET bromodomain inhibitors act, among others, by inhibiting NF-κB signaling and have already shown their potential in increasing therapeutic efficacy in MPN and might be particularly effective in SMF [52]. Surprisingly, MAPK and PI3K signaling was downregulated compared to HCs. This might be explained by a CD34^+^-dependent activation of different MAPK (ERK, p38, JNK) or PI3K (classes I_A_ and I_B_) pathways compared to the datasets that were used for training of the PROGENy algorithms. Estrogen signaling that was significantly downregulated regulates HSPC survival, proliferation, cytokine-production, and self-renewal [53]. Activation of the estrogen receptor with tamoxifen induced apoptosis in JAK2V617F HSPCs [54]. Therefore, estrogen signaling might be downregulated as a pro-survival mechanism.

Furthermore, immune system–related and pro-inflammatory pathways were upregulated in all subtypes, particularly in PMF and SMF. JAK1/2- and STAT3/5-mediated inflammatory processes play an important role in the pathogenesis and progression of MPN [55]. Among the key players in MPN pathophysiology are clonal HSPCs, as well as inflammatory cytokines and their interplay with endothelial and mesenchymal stromal cells (MSCs) [56]. The pro-inflammatory signatures are corroborated by the activation of TNF and NF-κB signaling pathways, identified in our PROGENγ analysis, and the effectiveness of inflammation modulating drugs such as interferon-alpha (IFNa).

We further identified the induction of GO terms associated with inflammation in all four MPN entities. These ontologies were most significantly enriched in PMF and SMF. In contrast, only PV had significantly downregulated ontologies comprising RNA splicing pathways. Mutations in RNA splicing–associated genes such as *SRSF2*, *SF3B1*, and *U2AF1* are often observed in MPN suggesting a pathophysiological role [5]. *SRSF2* mutations are associated with poor prognosis in PMF and enriched in leukemic transformation. These data support the poor risk profile of *SRSF2* mutation in PV and ET patients.

In addition, we observed enrichment of oxidative stress-associated genes in all four entities. The aberrant activation of JAK2 induces DNA damage by cell cycle–induced DNA damage in the hematopoietic stem cell compartment [57]. The frequency of these damaging events that lead to genomic instability is further increased by the accumulation of reactive oxygen species (ROS) [58]. ROS can stabilize HIF-1α and induce aberrant HIF signaling accompanied by enhanced glycolysis that both were identified as potential targets in MPN [59, 60]. On the other hand, excessive ROS levels induce apoptosis leading to the activation of NRF2 as a regulator of the cellular resistance to oxidative stress in CD34^+^ MPN cells. In contrast, downregulation of NRF2 signaling was reported in MPN patient-derived whole blood cells fueling the expansion of the hematopoietic progenitor pool [61]. We hypothesize a different role of NRF2 in CD34^+^ MPN cells as NRF2 is involved in the regulation of HSPC function by increasing quiescence and self-renewal [62].

A set of deregulated genes was identified that, beyond their putative role in MPN pathogenesis, may also have prognostic and therapeutic significance. *AREG* was downregulated in the CD34^+^ fraction of all four MPN entities and unfractionated BM from PMF patients. Although its molecular function in the context of MPN remains to be elucidated, it might serve as a molecular disease marker. The downregulation of *DNTT*, which is implicated in B cell development, might be indicative of the shift of HSPC fate towards the myeloid lineage.

We also identified several upregulated genes that might contribute to the pathogenesis of MPN such as the pro-inflammatory alarmins *S100A8* and *S100A9*. Both proteins are TLR4 ligands that activate NF-κB and induce the secretion of pro-inflammatory cytokines [63]. In MPN, the S100A8/A9-induced erythroid differentiation block might contribute to the high risk of leukemic transformation in PMF and SMF, whereas its expression is considerably lower in ET and PV [64]. Strikingly, the S100A8/S100A9 inhibitor tasquinimod significantly ameliorated the MPN phenotype and fibrosis in a JAK2V617F mouse model [35]. Furthermore, increased S100A8/S100A9 expression seems to play a central role in the telomere-associated inflammatory environment found in CML [65]. CYBB/NOX2 that was upregulated in all subtypes is a super-oxide-generating enzyme that interacts with S100A8/9 and might contribute to elevated ROS levels in MPN patients promoting the acquisition of additional mutations and the stabilization of HIF-1α [60, 66]. Elevated levels of S100A10 were reported to attenuate the pro-apoptotic effects of BAD [67]. Furthermore, it contributes to coagulopathy in AML by increasing the affinity of tPa for plasminogen leading to hyperfibrinolysis, which is associated with bleeding [68]. S100A12 is expressed in several tumors mediating proliferation, invasion, and migration [69].

TIMD4 that was upregulated in all subtypes was observed to induce T cell exhaustion in cancer and promote colorectal cancer by activating angiogenesis and recruitment of tumor-associated macrophages [70, 71]. In PMF, TIMD4 was reported to recruit monocytes/macrophages to the BM [72]. VCAM1, which was also elevated in our GEA, contributes to the clinical phenomena of thrombosis and extramedullary hematopoiesis occurring in MPN patients [73].

RT-qPCR analysis of these genes in unfractionated BM-derived cells revealed that their differential regulation is mainly restricted to CD34^+^ cells underlining the potential implication of these marker genes specifically within more primitive populations. This was particularly prominent for *S100A12* that was strongly upregulated in the CD34^+^ compartment but downregulated in unfractionated BM in all subtypes. We also identified potential marker genes for a transition from ET or PV to SMF (such as *CLEC1B*, *NMU*, or *DACH1*) that were strongly deregulated only in SMF. Within the top 10 up- and downregulated genes, we also observed elevated expression of *MTSS1* that is suppressed in high-risk AML but increased in patients with better clinical outcome [41].

Nevertheless, limitations of our study need to be discussed as well. The sample size of the SMF group is relatively small and all-female. The mutational landscape in the ET group is heterogeneous combining *JAK2-*, *CALR-*, *MPL-*mutated, and triple-negative patients possibly leading to a bias in the gene expression patterns between the different subtypes.

In summary, our investigation has demonstrated that CD34^+^ cells derived from PMF and SMF patients differ from ET and PV patients in the expression of single genes and the activation of signaling pathways. The different MPN subtypes are based on the mutual activation of the JAK-STAT pathway. Although this explains similarities such as the 47 jointly deregulated genes and a general activation of inflammatory signaling pathways, we observed differences between the subtypes that could contribute to modeling the disease transition from early to late stage MPN. Furthermore, several of these genes and pathways might constitute potential disease markers or candidates for targeted therapy, especially those genes deregulated only in the CD34^+^ fraction. Noteworthy is the strong activation of inflammatory pathways confirming that anti-inflammatory drugs such as ruxolitinib or BET bromodomain protein inhibitors or disease-modulating agents such as IFNa constitute a promising approach in Ph^−^ MPN, especially in later stages.

## Supplementary Information

Below is the link to the electronic supplementary material.

Supplementary file1 (DOCX 4375 kb)

Supplementary file2 (XLSX 243 kb)

## Data Availability

Microarray data have been deposited at GEO under accession number GSE174060.
