# Supplementary material for: Early and late stage MPN patients show distinct gene expression profiles in CD34+ cells
Source: Ann Hematol. 2021 Aug 14;100(12):2943–56. doi: 10.1007/s00277-021-04615-8 (PMC8592960; doi:10.1007/s00277-021-04615-8)
Supplement: Supplementary file 1 — Supplementary file1 (DOCX 4375 kb) [file 277_2021_4615_MOESM1_ESM.docx]

**Early and late stage MPN patients show distinct gene expression profiles in CD34^+^ cells**

Running title: Gene expression in MPN patient-derived CD34^+^ cells

Julian Baumeister,^1,2^ Tiago Maié,^2,3^ Nicolas Chatain,^1,2^ Lin Gan,^4^ Barbora Weinbergerova,^5^ Marcelo A. S. de Toledo,^1,2^ Jörg Eschweiler,^6^ Angela Maurer,^1,2^ Jiri Mayer,^5^ Blanka Kubesova,^5^ Zdenek Racil,^7^ Andreas Schuppert,^2,8^ Ivan Costa,^2,3^ Steffen Koschmieder,^1,2^ Tim H. Brümmendorf,^1,2^ Deniz Gezer^1,2^

*^1^Department of Hematology, Oncology, Hemostaseology, and Stem Cell Transplantation, Faculty of Medicine, RWTH Aachen University, Aachen, Germany; ^2^Center for Integrated Oncology Aachen Bonn Cologne Duesseldorf (CIO ABCD), Germany; ^3^Institute for Computational Genomics, RWTH Aachen University, Aachen, Germany; ^4^ IZKF Genomics Core Facility, RWTH Aachen University Medical School, Aachen, Germany; ^5^Department of Internal Medicine, Hematology and Oncology, Masaryk University and University Hospital Brno, Brno, Czech Republic; ^6^Department of Orthopedic Surgery, University Hospital RWTH Aachen, Aachen, Germany; ^7^Institute of Hematology and Blood Transfusion, Prague, Czech Republic; ^8^ Joint Research Center for Computational Biomedicine, RWTH Aachen, Aachen, Germany*

**Corresponding Author**

Dr. Deniz Gezer, Department of Hematology, Oncology, Hemostaseology and Stem Cell Transplantation, Faculty of Medicine, RWTH Aachen University, Pauwelsstr. 30, D‑52074 Aachen, Germany, Phone: +49-241-8037225, E-mail: dgezer@ukaachen.de

# Supplemental Figures


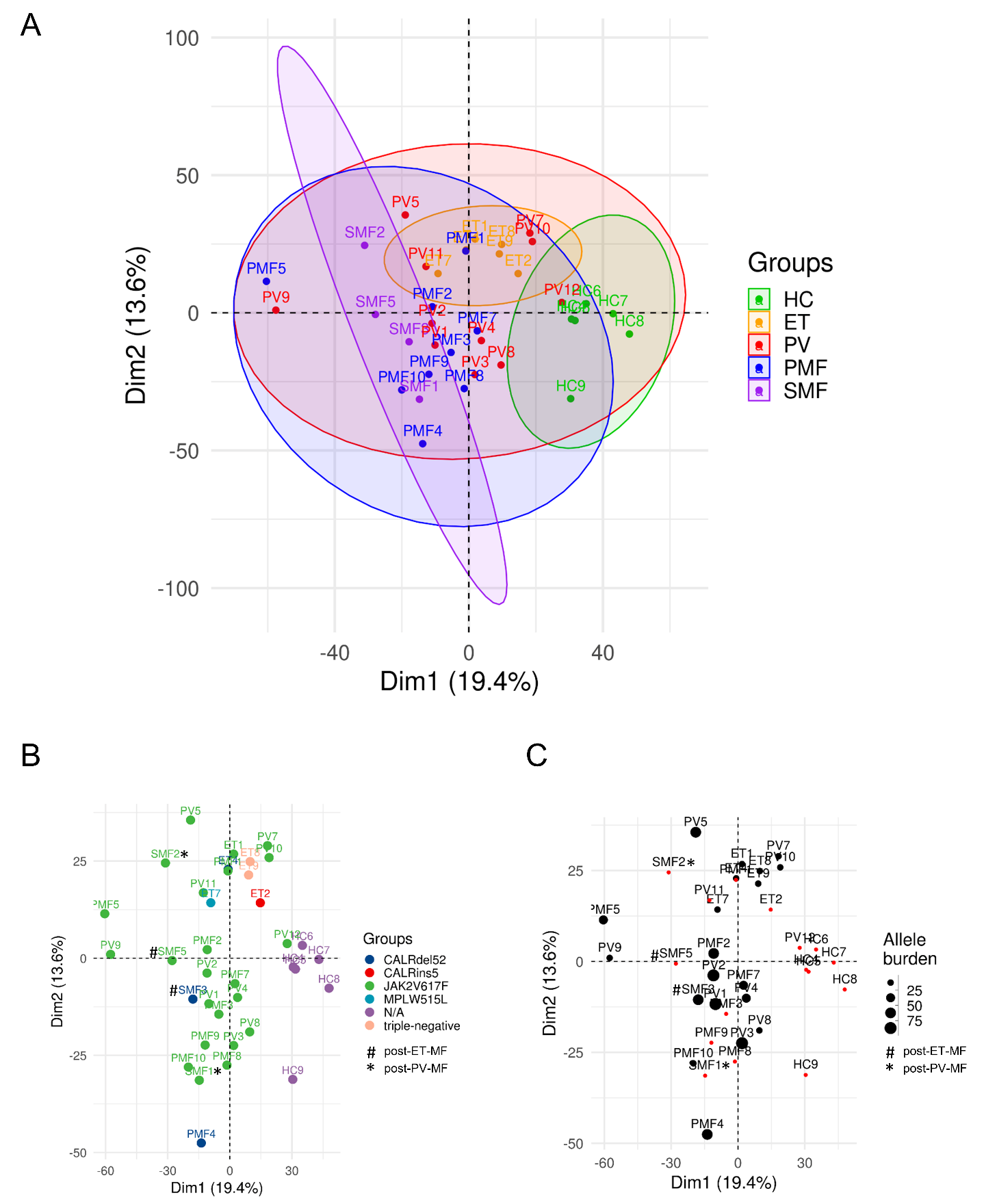


**Supplemental Figure 1: Principal component analysis (PCA) including driver mutations and allele burden.** (A) PCA with distribution of the samples within the different entities. (B) PCA including the driver mutations JAK2V617F (green), CALRdel52 (blue), CALRins5 (red), MPLW515L (cyan) or triple-negative negative patients (beige) or HCs (violet). (C) PCA including driver mutation allele burden (AB). AB is given as data point size in %. HCs and patients with missing data are depicted in red.


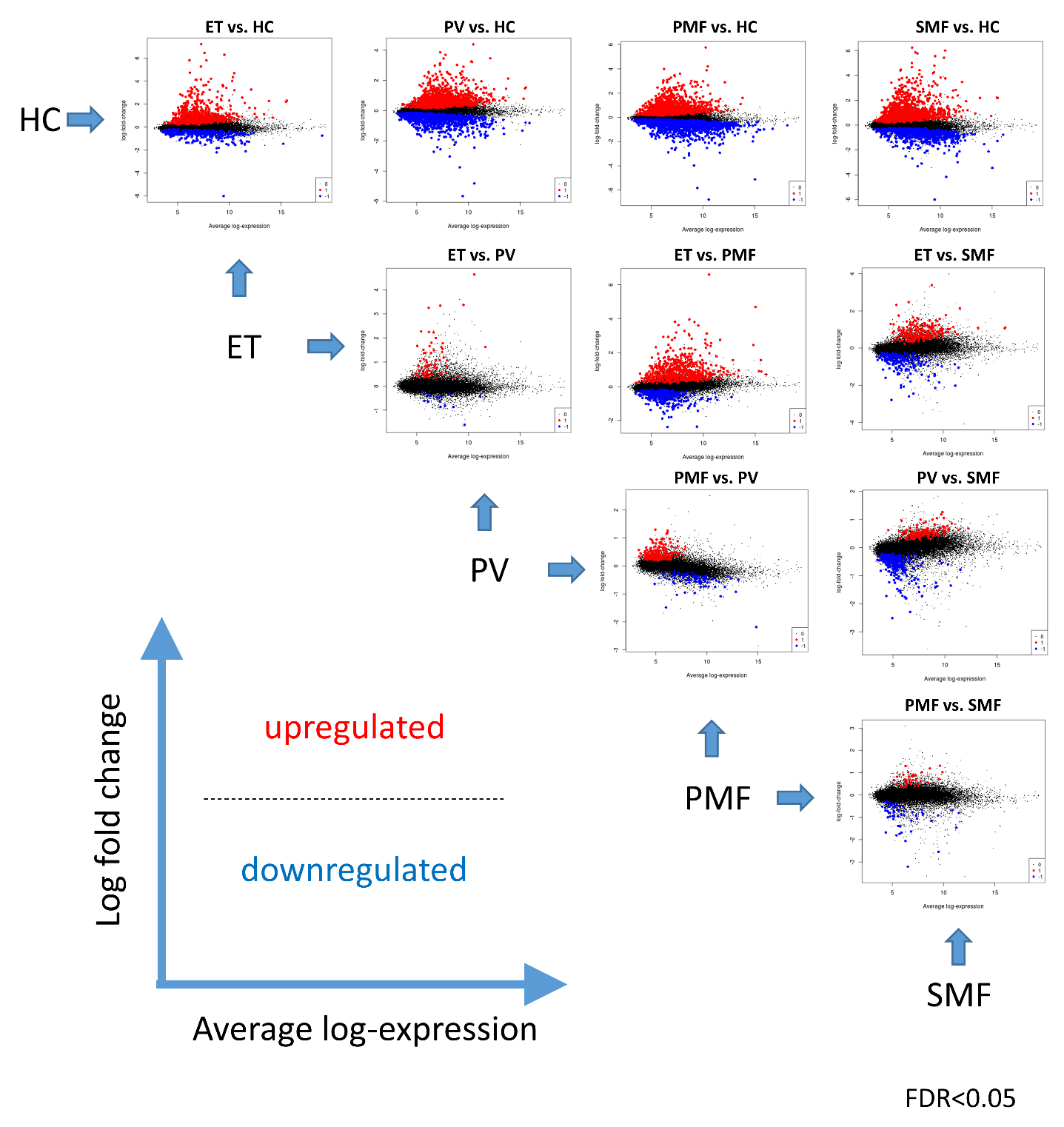


**Supplemental Figure 2: Differentially regulated genes between the different MPN subtypes.** Data points represent individual genes, plotted by log2 fold change (y-axis) and average log-expression (x-axis). Genes with an FDR>0.05 are highlighted in red (upregulated) or blue (downregulated). ET, essential thrombocythemia; HC, healthy controls; PMF, primary myelofibrosis; PV, polycythemia vera; SMF, secondary myelofibrosis.


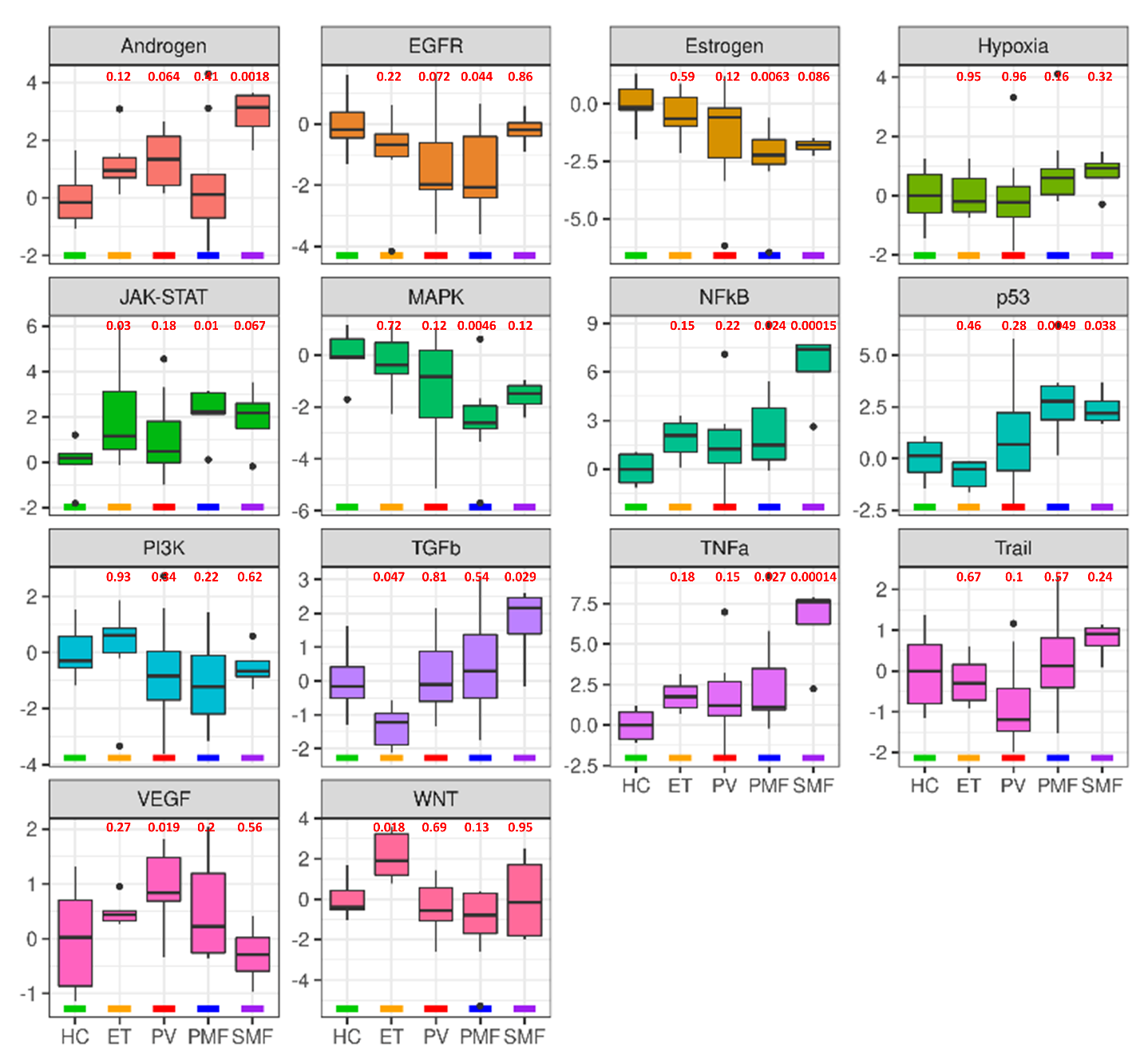


**Supplemental Figure 3: PROGENγ activity scores in CD34^+^ enriched MNCs from ET, PV, PMF, SMF patients and HC.** PROGENγ analysis of androgen, EGFR, estrogen, hypoxia, JAK/STAT, MAPK, NF-κB, p53, PI3K, TGFβ, TNFα, Trail, VEGF and WNT signaling pathways in patient or healthy donor derived CD34^+^ cells, arranged by group (HC, ET, PV, PMF and SMF).Values are normalized in respect to HC. P-values, indicated in red, were calculated by t-test (* p<.05, ** p<.01, *** p<.001). ET, essential thrombocythemia; HC, healthy controls; PMF, primary myelofibrosis; PV, polycythemia vera; SMF, secondary myelofibrosis.


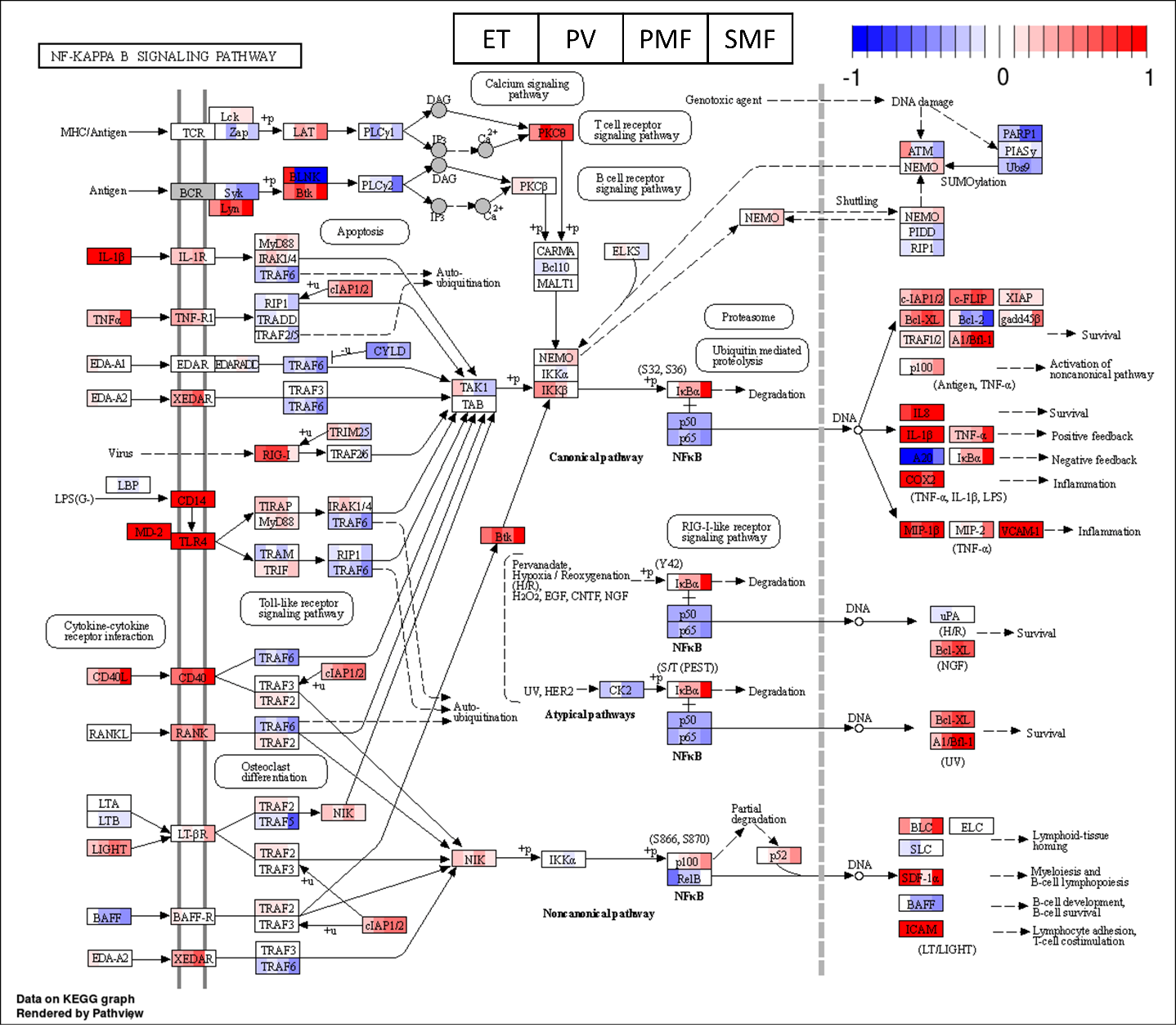


**Supplemental Figure 4: NF-κB signaling pathway.** KEGG analysis of canonical, noncanonical and atypical NF-κB signaling pathways in ET, PMF, PV and PMF, compared with HC. Upregulated genes are highlighted in red and downregulated genes in blue in the respective boxes of each gene (max. fold change=1). ET, essential thrombocythemia; HC, healthy controls; NF-κB, nuclear factor κB; PMF, primary myelofibrosis; PV, polycythemia vera; SMF, secondary myelofibrosis.


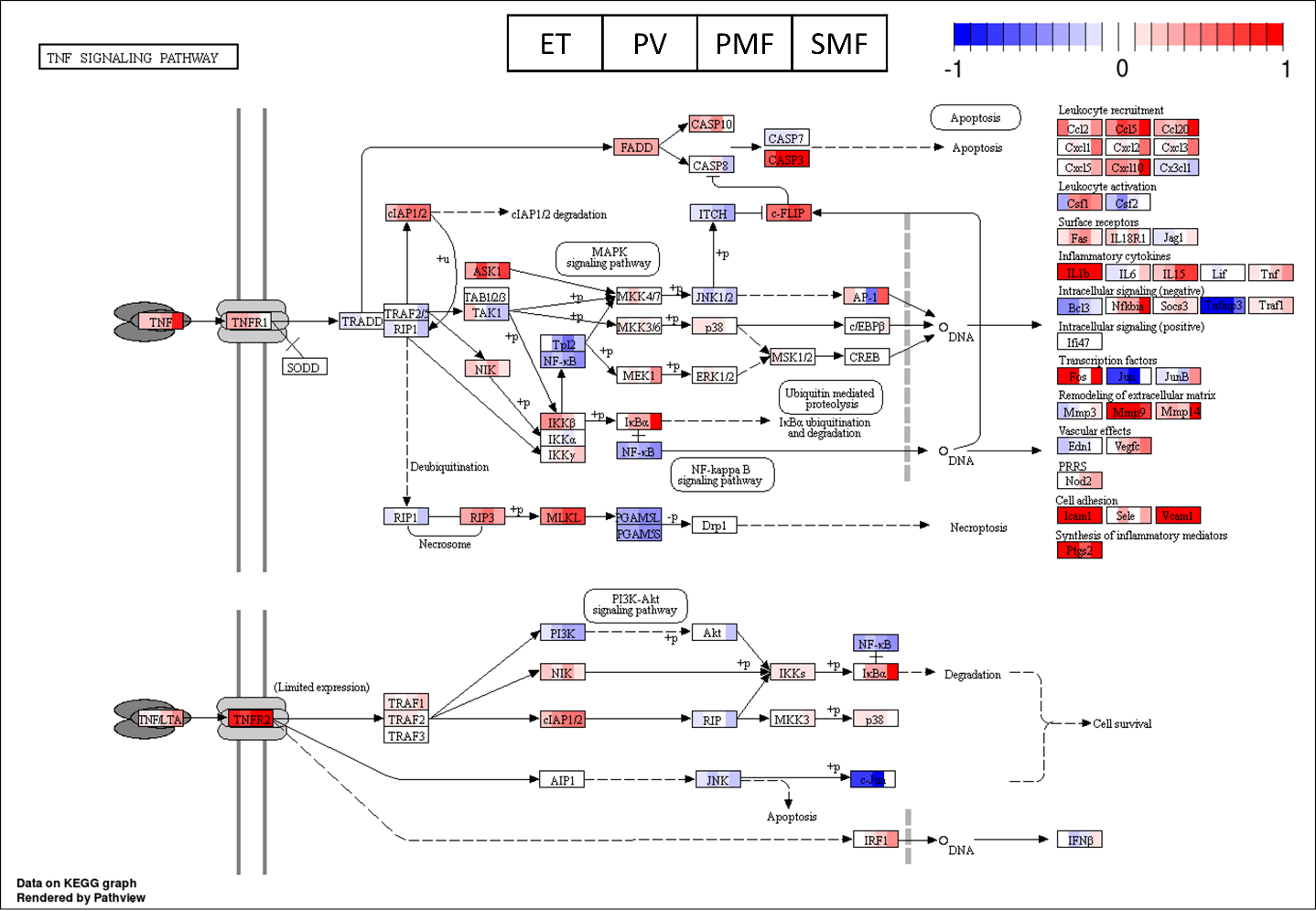


**Supplemental Figure 5: TNF signaling pathway.** KEGG analysis of the TNF signaling pathway in ET, PMF, PV and PMF, compared with HC. Upregulated genes are highlighted in red and downregulated genes in blue in the respective boxes of each gene (max. fold change=1). ET, essential thrombocythemia; HC, healthy controls; PMF, primary myelofibrosis; PV, polycythemia vera; SMF, secondary myelofibrosis; TNF, tumor necrosis factor.


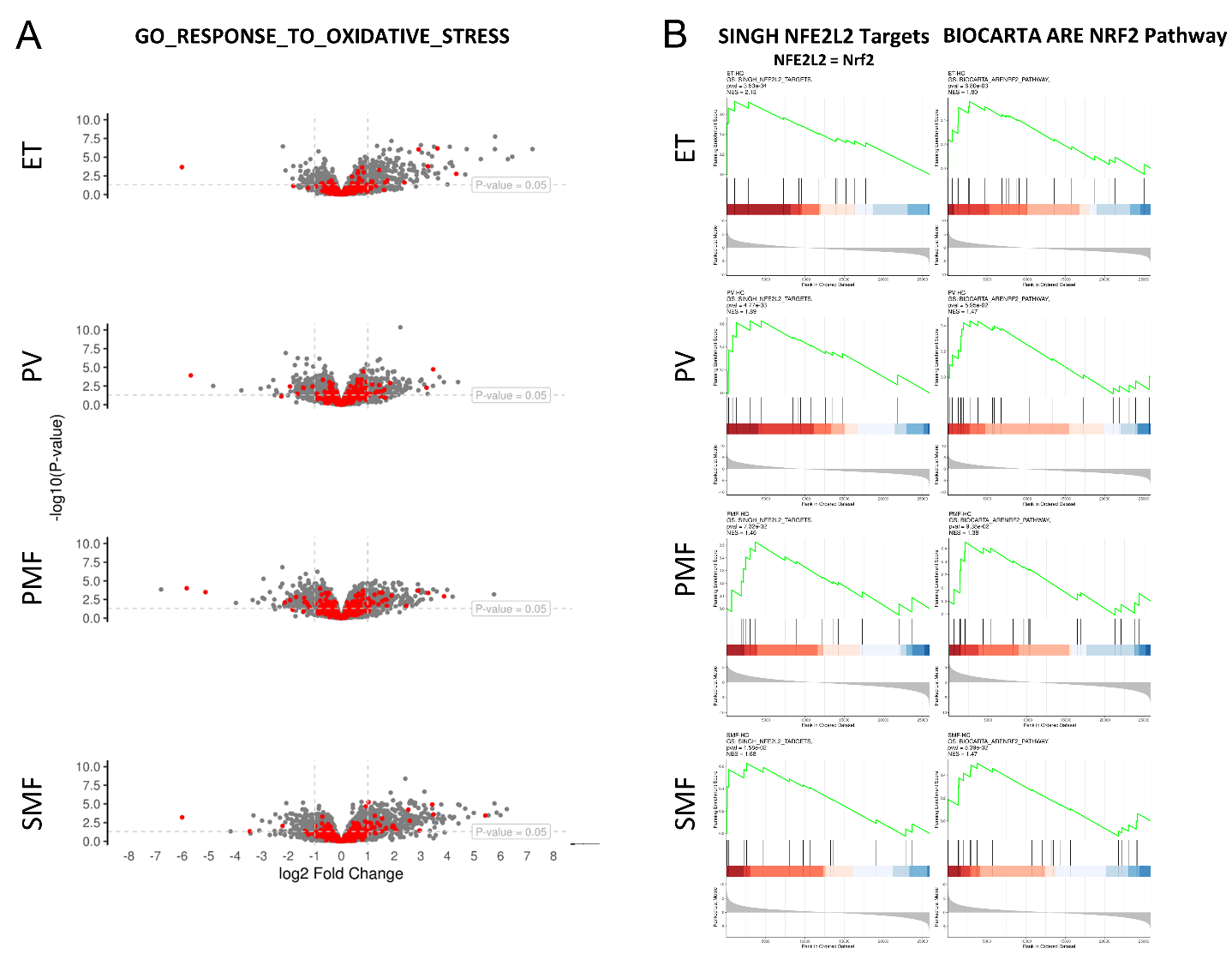


**Supplemental Figure 6: Oxidative stress response genes in MPN patient derived CD34^+^ cells.** (A) Volcano plot highlighting genes of the gene ontology dataset GO_RESPONSE_TO_OXIDATIVE_STRESS in red color in CD34^+^ cells from ET, PV, PMF and SMF patients vs. HC. (B) Gene set enrichment analysis (GSEA) of NRF2 target genes by Singh et al.^1^ and the BioCarta ARE NRF2 pathway. Upregulated genes are highlighted in red and downregulated genes in blue. The y-axis indicates the enrichment of genes in the respective pathway. ARE, antioxidant response element; ET, essential thrombocythemia; HC, healthy controls; NRF2, nuclear factor erythroid 2-related factor 2; PMF, primary myelofibrosis; PV, polycythemia vera; SMF, secondary myelofibrosis.


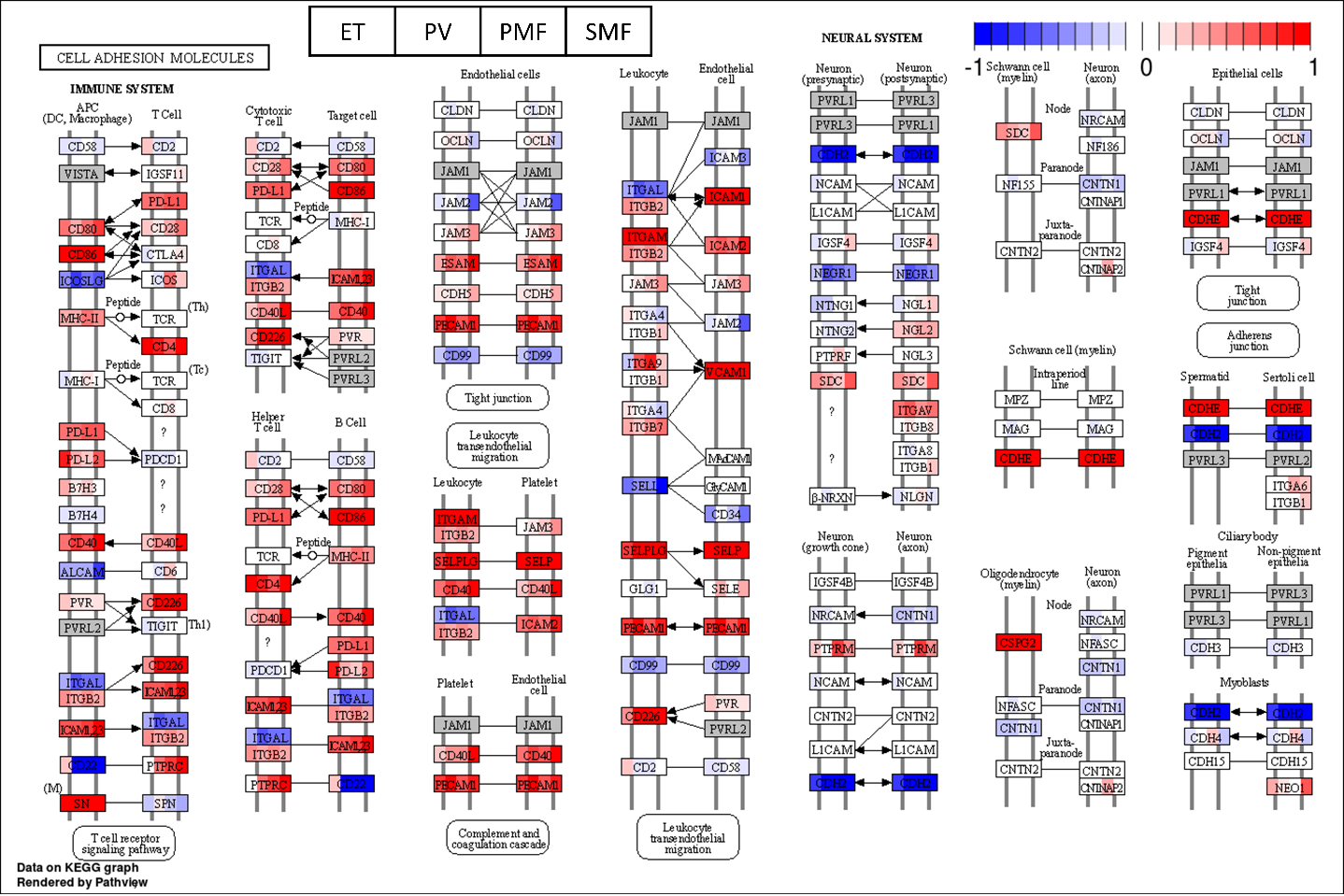


**Supplemental Figure 7: Expression of cell adhesion molecules.** KEGG analysis of cell adhesion molecules in ET, PMF, PV and PMF, compared with HC. Upregulated genes are highlighted in red and downregulated genes in blue in the respective boxes of each gene (max. fold change=1). ET, essential thrombocythemia; HC, healthy controls; PMF, primary myelofibrosis; PV, polycythemia vera; SMF, secondary myelofibrosis.


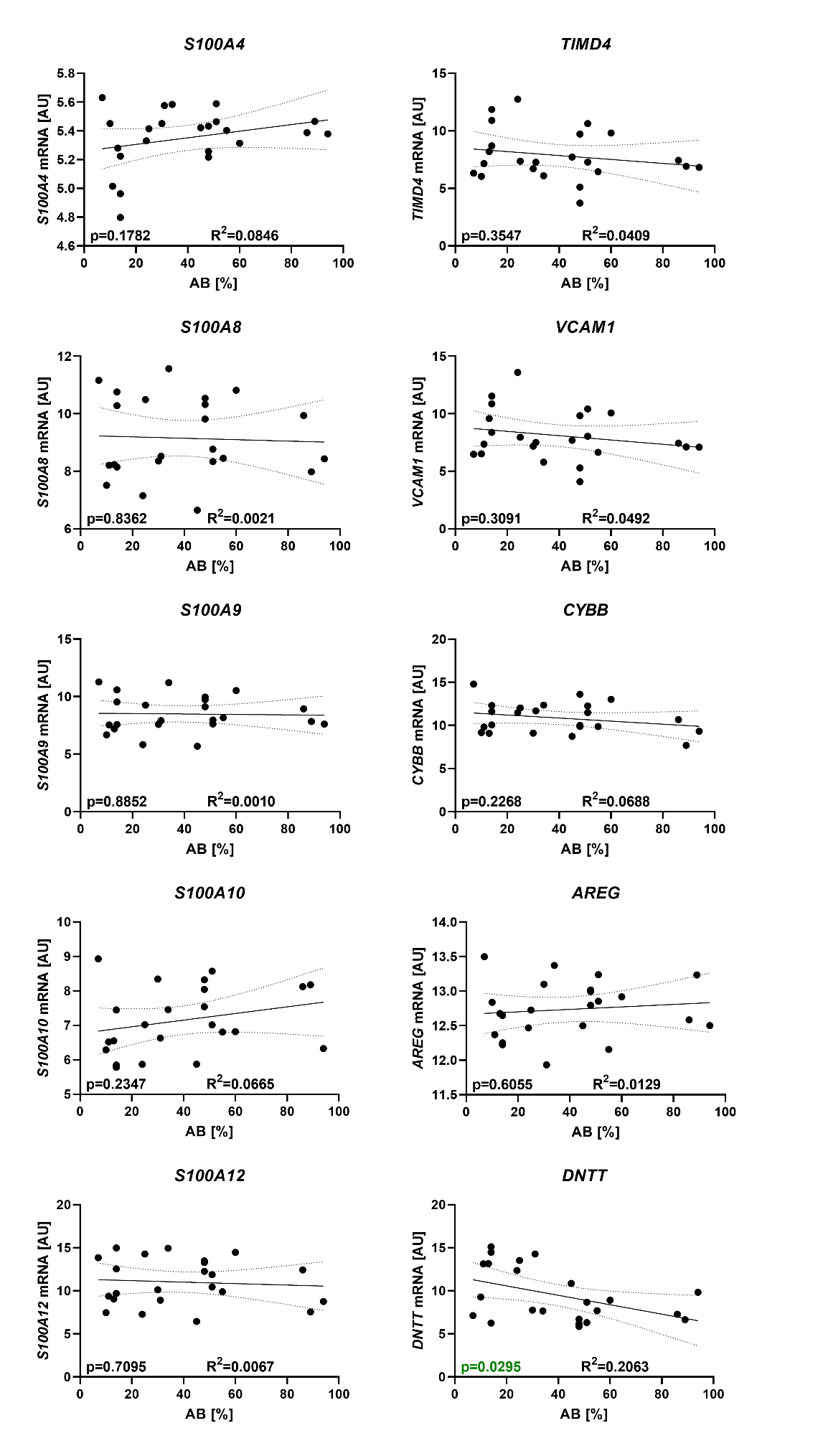


**Supplemental Figure 8: Correlation of gene expression with JAK2V617F allele burden.**  Correlation of absolute gene expression (AU=arbitrary unit) of AREG, DNTT, S100A4, S100A8, S100A9 and S100A12 vs. allele burden. Standard line was interpolated without special handling of outliers and 95% confidence bands.

1. Singh A, Boldin-Adamsky S, Thimmulappa RK, Rath SK, Ashush H, Coulter J*, et al.* RNAi-mediated silencing of nuclear factor erythroid-2-related factor 2 gene expression in non-small cell lung cancer inhibits tumor growth and increases efficacy of chemotherapy. *Cancer Res* 2008 Oct 1; **68**(19)**:** 7975-7984.
